# Facilitating access to medicines and continuity of care for Ukrainian refugees: exceptional response or the promise of more inclusive healthcare for all migrants?

**DOI:** 10.1136/bmjgh-2022-010327

**Published:** 2022-08-25

**Authors:** Saleh Aljadeeah, Joris Michielsen, Raffaella Ravinetto, Sally Hargreaves, Veronika J. Wirtz, Oliver Razum, Federico Gobbi, Karina Kielmann

**Affiliations:** 1 Department of Public Health, Institute of Tropical Medicine, Antwerpen, Belgium; 2 Institute for Infection and Immunity, St George’s, University of London, London, UK; 3 Department of Global Health, Boston University Scool of Public Health, Boston, Massachusetts, USA; 4 Department of Epidemiology & International Publich Health, School of Public Health, Bielefeld University, Bielefeld, Germany; 5 Department of Infectious/Tropical Diseases and Microbiology, IRCCS Sacro Cuore-Don Calabria, Negrar di Valpolicella, Verona, Italy

**Keywords:** Public Health

The Ukrainian refugee crisis has received prompt attention not only from concerned citizens outside Ukraine and the United Nations agencies but also from the European Commission (EC). For the first time, the Temporary Protection Directive 2001/55/EC was activated, enabling immediate access to healthcare for Ukrainian citizens fleeing their country.^1^ In European Union (EU) member states, several initiatives were quickly mobilised; municipalities and local health systems received tailored guidance on how to address health needs of Ukrainian refugees, and platforms were created to inform them on their rights under this Directive.^2^ Discussions went beyond temporary measures, calling on health systems to address the burden of long-term chronic diseases and multimorbidities for Ukrainian patients, requiring continuity of care including sustained access to medicines.^3^ In a joint appeal, for instance, the European Centre for Disease Prevention and Control, the WHO and the European Association for the Study of Liver call for free and sustained treatment for hepatitis B and/or C including antiviral medicines.^4^

These are very welcome developments. However, this Directive is not applied to non-Ukrainians fleeing from the same war, nor—looking back over the past decade(s)—was it applied to those fleeing from other conflicts. Refugees who reached the EU in 2014 and 2015 were often excluded from mainstream health and vaccination systems and were stranded in camps where they depended on healthcare provided by non-governmental organizations (NGOs).^5^

To date, fragmented access to healthcare remains a reality for many migrants in Europe, yet little is known about how migration impacts on continuity of care.^6^ Essential medicines are a public good and a cornerstone of comprehensive care packages. Their availability to those in need is also a strong indicator of health systems performance and health equity.^7^ But migrants in Europe may face system-related restrictions as well as financial barriers, language and cultural barriers.^8^ These barriers negatively affect access to medicines, medicine utilisation, treatment adherence and patient safety and are particularly relevant for conditions requiring long-term management.^8^ Even when medicines are available and adequately prescribed and reimbursed, concerns around safety, livelihood and stress related to the request for asylum may outweigh the capacity to manage chronic conditions.^8 9^ The limited qualitative research available on the experience of refugees provides stark illustrations of the challenges: one recent study from Norway suggests that complex conditions were inadequately addressed in this group, partly because of limitations in the time available for consultation and dispensation, and the interpreters’ capacity. As expressed poignantly by a 50-year-old participant: “it’s not that if you have a headache, well then you get a pill … this is not where the problem lies, but it is about having special diseases and complex problems and if the doctor does not understand you properly then you will not get the right treatment”.^10^ Untreated chronic conditions can impede on other steps to successful integration, for example, attendance of language classes,^8^ and increase the long-term healthcare costs for individuals, families and the host country.^11^

Further, providing continuity of care for migrants with conditions requiring long-term treatment may pose particular challenges when the required medicines are in short supply in the EU. Multidrug-resistant (MDR) tuberculosis (TB) provides a strong case. Reportedly, 1400 adults and 160 children out of those who fled Ukraine need TB treatment and approximately a third of them require treatment for MDRTB. Some of the medicines needed for this indication are in limited supply in EU countries, or they are neither registered nor licenced, or they are licensed but too costly.^12^

These examples indicate the short-sightedness of policies that do not take the reality of severe, complex and/or chronic conditions into account. There is an urgent need to bridge the short-term emergency response and medium-to-long-term actions, to integrate migrant populations in the mainstream European health systems which ensure continuous access to medicines.^6^ Research can shed light on the health needs of migrants including those living with chronic diseases and complex multimorbidities and can inform policies and strategies for inclusive, sustainable, equitable and compassionate care. We need to better understand the implementation of legal and policy provisions for access to health and essential medicines and how these affect migrants’ pathways to care, both within European countries and across transnational networks. This will help European health policy-makers to adapt health policies and the systems that implement these towards longer-term and more equitable perspectives on sustained provision of care for all migrants, which goes beyond emergency care.

## Data Availability

There are no data in this work.

## References

[1] European Commission. Migration and Home Affairs, 2022. Available: https://home-affairs.ec.europa.eu/policies/migration-and-asylum/common-european-asylum-system/temporary-protection_en [Accessed 11 Jul 2022].

[2] European Commission. Fleeing Ukraine: Healthcare, 2022. Available: https://ec.europa.eu/info/strategy/priorities-2019-2024/stronger-europe-world/eu-solidarity-ukraine/eu-assistance-ukraine/information-people-fleeing-war-ukraine/fleeing-ukraine-healthcare_en#access-to-healthcare-in-the-eu [Accessed 11 Jul 2022].

[3] Cullinan K. As Ukrainians Flee, WHO stresses importance of lifesaving Ncd care for refugees and migrants. Available: https://healthpolicy-watch.news/ncd-care-for-refugees/ [Accessed 09 Jul 2022].

[4] European Centre for Disease Prevention and Control. Joint statement: ensuring high-quality viral hepatitis care for refugees from Ukraine, 2022. Available: https://www.ecdc.europa.eu/en/news-events/joint-statement-ensuring-high-quality-viral-hepatitis-care-refugees-ukraine [Accessed 15 Jul 2022].

[5] Kondilis E, Papamichail D, McCann S, et al. The impact of the COVID-19 pandemic on refugees and asylum seekers in Greece: a retrospective analysis of national surveillance data from 2020. EClinicalMedicine 2021;37:100958. 10.1016/j.eclinm.2021.100958 34258570 PMC8256175

[6] World Health Organization (WHO). Continuum of care for noncommunicable disease management during the migration cycle. Geneva: World Health Organization, 2022. 35536925

[7] Wirtz VJ, Hogerzeil HV, Gray AL, et al. Essential medicines for universal health coverage. The Lancet 2017;389:403–76. 10.1016/S0140-6736(16)31599-9 PMC7159295 27832874

[8] Aljadeeah S, Wirtz VJ, Nagel E. Barriers to accessing medicines among Syrian asylum seekers and refugees in a German federal state. Int J Environ Res Public Health 2021;18:519. 10.3390/ijerph18020519 PMC7827681 33435189

[9] Hadgkiss EJ, Renzaho AMN. The physical health status, service utilisation and barriers to accessing care for asylum seekers residing in the community: a systematic review of the literature. Aust Health Rev 2014;38:142–59. 10.1071/AH13113 24679338

[10] Haj-Younes J, Abildsnes E, Kumar B, et al. The road to equitable healthcare: a conceptual model developed from a qualitative study of Syrian refugees in Norway. Soc Sci Med 2022;292:114540. 10.1016/j.socscimed.2021.114540 34763966

[11] Bozorgmehr K, Razum O. Effect of restricting access to health care on health expenditures among Asylum-Seekers and refugees: a quasi-experimental study in Germany, 1994-2013. PLoS One 2015;10:e0131483. 10.1371/journal.pone.0131483 26201017 PMC4511805

[12] Ravelo JL. WHO, stop TB seek permits for MDR-TB medicines for Ukraine refugees, 2022. Available: https://www.devex.com/news/who-stop-tb-seek-permits-for-mdr-tb-medicines-for-ukraine-refugees-102918 [Accessed 11 Jul 2022].

